# Guillain–Barre syndrome after myocardial infarction: a case report and literature review

**DOI:** 10.1186/s12872-023-03261-4

**Published:** 2023-05-01

**Authors:** Pu-yuan Wen, Xian-wen Chen, Min Zhang, Wen-zheng Chu, Hong-liang Wu, Chao Ren

**Affiliations:** 1 Department of Neurology, Affiliated Yantai Yuhuangding Hospital of Qingdao University, Yantai, Shangdong 264000 China; 2 Department of Neurology, The First Affiliated Hospital of Anhui Medical University, Hefei, Anhui 230000 China

**Keywords:** Guillain–Barre syndrome, Myocardial infarction, Immune-mediated response, Surgery, Streptokinase

## Abstract

**Background:**

Guillain–Barre syndrome after myocardial infarction occurs infrequently, and its occurrence following percutaneous coronary intervention is extremely rare. Due to the high mortality rate of myocardial infarction and the disability of Guillain–Barre syndrome, early identification of Guillain–Barre syndrome after myocardial infarction and early intervention can decrease the mortality rate, lead to early recovery, and provide a better outcome.

**Case presentation:**

Herein, we reported a rare case of Guillain–Barre syndrome after myocardial infarction treated with percutaneous coronary intervention. The patient was a 75-year-old woman from China who was admitted to hospital due to sudden loss of consciousness. Electrocardiography showed acute myocardial infarction in the right ventricle and inferior and posterior walls. The patient underwent emergency percutaneous intervention of the posterior collateral artery of the right coronary artery. Soon after, her condition worsened resulting in limb weakness and numbness. Unfortunately, she continued to develop respiratory failure, and treated with intravenous immunoglobulin and ventilator-assisted breathing. A physical examination showed hypotonia of all four limbs, complete quadriplegia, bulbar palsy, dysarthria, and tendon areflexia. Serum immunoglobulin (Ig) G anti-ganglioside antibody analysis was positive with anti-GT1a antibodies (+ +), anti-GM1 antibodies ( +), anti-GM2 antibodies ( +), and anti-GM4 antibodies ( +), and he was diagnosed with Guillain–Barre syndrome after myocardial infarction. She was discharged due to poor response to treatment. The patient died two days after being discharged.

**Conclusions:**

Myocardial infarction and/or percutaneous coronary intervention may activate immune-mediated response and cause severe complications. Clinician should be alert to Guillain–Barre syndrome after myocardial infarction and/or percutaneous coronary intervention.

**Supplementary Information:**

The online version contains supplementary material available at 10.1186/s12872-023-03261-4.

## Background

Guillain–Barre syndrome (GBS) is an autoimmune peripheral neuropathy with the main clinical manifestation being acute symmetrical flaccid paralysis. GBS has a very low incidence of 1/100 000 to 2/100 000 per year worldwide [1] and increases by 20% for every ten-year increase in age, that of incidence in males is about 1.5 times higher than in females [2]. It has been reported that east Asia had lower incidence of GBS than North America and Europe, with 0.67 cases per100000 person-years in China [3].

The pathological characteristics of the syndrome are hyperaemia and oedema of the nerve root, ganglia, and peripheral nerves. Local perivascular inflammatory cell infiltration and nervous demyelinating changes may even result in axonal degeneration. The syndrome is indicated by multiple lesions in the nerve root and peripheral nerves, and a cerebrospinal fluid (CSF) examination reveals albumino-cytologic dissociation. The diagnosis of GBS mainly depends on the typical clinical features of symmetric flaccid paresis with decreased or absent reflexes, nerve conduction studies (NCS), and albumin-cytological dissociation of cerebrospinal fluid (CSF) and serum anti-ganglioside antibodies detection all supporting the diagnosis. At present, intravenous immunoglobulin and plasmapheresis are the only recognized treatment methods for GBS. After timely treatment, most patients generally have a good prognosis, but severe cases presenting respiratory paralysis often require mechanical ventilation, and eventually poor prognosis or death due to infection and other complications. GBS generally occurs after infections, mostly by Epstein–Barr, Campylobacter jejuni, cytomegalovirus, and influenza viruses, but can also be triggered by surgery, vaccination [4]. The association between the vaccine and GBS is still unclear, with less than 1 case of GBS occurring per million people with each vaccine [5]. Gensicke et al. [6] showed that the risk of GBS occurring within 6 weeks after a surgery was 13.1 times that of the general population.

The occurrence of GBS after myocardial infarction (MI) is rare, with only 15 reported cases [7–20]. Herein, we describe a rare case of GBS after acute MI treated with percutaneous coronary intervention (PCI). By analyzing the clinical characteristics and possible pathogenesis of these 16 cases, it indicated that MI and/or PCI may activate immune response, and clinician should be alert to the occurrence of GBS after MI, especially in MI treated with PCI.

The patient’s next to kin (her daughter) gave her written informed consent to participate.

## Case presentation

**Fig. 1:**
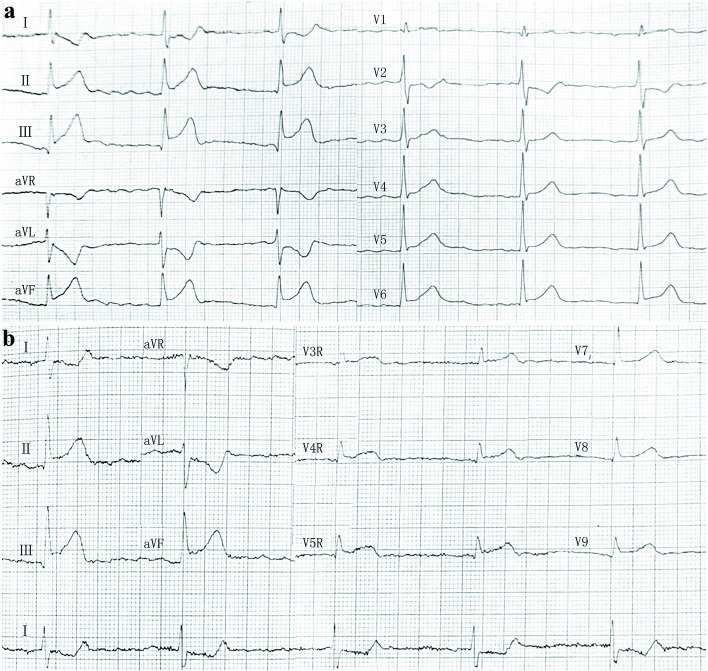
Electrocardiogram showed acute myocardial infarction in the right ventricle and inferior and posterior walls

A 75-year-old Chinese female patient was admitted to a local hospital for sudden loss of consciousness and limb weakness. Four days prior to admission the patient had suddenly lost consciousness while eating. After taking 0.6 mg nitroglycerin under the tongue for about 10 min, her consciousness was restored, but she continued to feel chest tightness and discomfort. An electrocardiogram upon admission showed ST-segment elevation in leads II, III, aVF, V_3R_-V_5R_ and V7-V9, reciprocal ST-segment depression in leads I and aVL, and an IIIA-V block (Fig. 1a, b), suggesting inferior, anterior, and posterior MI. Coronary angiography revealed 90% stenosis of the proximal segment of the anterior descending branch, occlusion of the distal branch of the posterior left ventricle and multiple branches, and diffuse lesions in the middle segment of the right crown, with the most severe stenosis reaching 85%. The middle segment of the right coronary artery was suspected to be the affected vessel as autolysis of a thrombus was noted, and a partial thrombus had spread to its distal branches (See Supplementary Figure S1.a, Additional File 1). The patient underwent emergency percutaneous intervention in middle segment of the right coronary artery (See Supplementary Figure S1.b, Additional File 1). Twenty hours prior to admission, the patient presented with sub-acute onset of bilateral upper limb weakness (Medical Research Council (MRC) sum score was 54), which gradually progressed from the distal to proximal end, and numbness. This was followed by weakness in bilateral lower limbs, which showed a similar progression with an impairment in the ability to hold urine. No obvious abnormality was found on cranial computed tomography (CT); thereafter, the patient was transferred to our hospital. The patient had previous histories included hypertension, type 2 diabetes, atrial fibrillation, and cerebral infarction without gastrointestinal infection and respiratory tract infection prior to the onset of MI. On clinical assessment, her ventricular rate was high (105 bpm) with atrial fibrillation rhythm; she had a blood pressure of 169/97 mmHg and temperature of 37.1 °C. A physical examination showed hypotonia of all four limbs, complete quadriplegia, superficial hypoesthesia in the extremities, bulbar palsy (dysarthria, decreased pharyngeal reflex, disappeared cough reflex), and tendon areflexia. Cerebellar system, fundus examinations and other cranial nerves showed no abnormality.

Her complete blood count, blood picture, C-reactive protein level, serum electrolyte levels, and hepatic and renal function were all normal. Her serum protein levels were as follows: total (6.5 g/dL), albumin (3.039 g/dL), and globulin (3.5 g/dL). Serum Mycoplasma pneumoniae antibody- immunoglobulin (Ig) M, EBV antibody to viral capsid antigen-IgM, EBV antibody to nuclear antigen of IgM, cytomegalovirus (CMV) antibody-IgM, and CMV pp65 antigen in peripheral blood leukocytes and human immunodeficiency virus antibody test results were all negative. B-type natriuretic peptide: 526.44 pg/mL (Normal 0–100 pg/mL); Procalcitonin: 1.69 ng/mL (Normal < 0.05 ng/mL). Serum IgG anti-ganglioside antibody test was positive with anti-GT1a antibodies (+ +), anti-GM1 antibodies ( +), anti-GM2 antibodies ( +), and anti-GM4 antibodies ( +). Repeated brain CT showed no abnormality. The family refused a lumbar puncture. Due to her critical condition, nerve conduction studies and electromyography could not be performed.

Later, she developed respiratory muscle weakness with low oxygen saturation and had trouble coughing and expectorating. This eventually progressed to respiratory failure, and she required endotracheal intubation and ventilator-assisted ventilation.

After diagnosis of GBS, the patient was treated with intravenous immunoglobulin (IVIg; a standard single IVIg dose [0.4 g/kg bodyweight/day]) and supportive care. Despite completing 5 consecutive days of IVIg therapy, her clinical symptoms did not improve. Later, the patient subsequently developed severe pneumonia, septic shock and rhabomyolysis. At the request of her family, she was discharged on the 23rd day of hospitalization. The patient died due to respiratory failure and circulatory collapse two days following discharge.

## Discussion

Occurrence of GBS after MI is rare, and in particular, it has never been reported a case of GBS after MI treated with PCI. Hence, it is essential to summarise and analyse the clinical features based on possible pathogenesis of past cases of GBS after MI reported in the literature, in order to be known deeply by clinician. By reviewing the 15 cases reported previously along with our case (Table 1), we found that the age of patients ranged from 46 to 75 years, and there was a male preponderance (14 men and 2 women); the time from MI to the occurrence of GBS ranged from 1 to 21 days. All 16 patients had diverse clinical manifestations, including paraesthesia (13 patients), facial paralysis (5 patients; 2 patients with unilateral facial paralysis and 3 with bilateral facial paralysis), autonomic dysfunction (3 patients), dysphagia (3 patients), and dyspnoea with tracheal intubation and mechanical ventilation (MV) (3 patients) (Table 2). Generally, the main cause of death in GBS patients is respiratory muscle paralysis, infection, hypotension, and severe arrhythmia. However, out of the 16 reported cases, death occurred in three; one patient died because of ventricular arrhythmia and two due to cardiopulmonary arrest. Ventricular arrhythmias might be a complication of MI or might be induced or aggravated by autonomic nerve damage during GBS. The coexistence of MI and GBS is more likely to lead to respiratory cardiac arrest. Therefore, early diagnosis of GBS after MI is extremely important.

**Table 1:** Published case reports of GBS after MI

First Author	Sex	Clinical manifestations of nervous system	Appearance of GBS (days)	Heart problems	Treatment for GBS	Treatment for MI	MV	Outcome
1 Eden et al. [7]	M	Paresthesia, quadriparesis	11	Acute inferior MI	Prednisone, physiotherapy	Streptokinase	NO	Muscle strength and deep-tendon normal
2 Leaf et al. [8]	M	Leg weakness, dyspnoea	9	Acute inferior MI. Right coronary artery occluded	N/A	Transluminal angioplasty, streptokinase	YES	Progressive recovery
3 McDonagh and Dawson [9]	M	Paresthesia, quadriparesis, areflexia	17	ST-segment elevation inferior MI	Physiotherapy	N/A	NO	Full recovery
4 McDonagh and Dawson, 1987 [9]	F	Paresthesia, papilloedema, areflexia	12	ST-segment elevation anterior MI	Physiotherapy	N/A	NO	Good return of motor function
5 Roquer et al. [10]	M	Quadriparesis, left facial paralysis, severe autonomic disfunction, dyspnoea	15	Acute anterior transmural MI	N/A	Streptokinase	YES	Good
6 Barnes et al. [11]	M	Paresthesia, bilateral facial paralysis, severe bulbar weakness, quadriparesis	21	MI	Plasmapheresis	Streptokinase	NO	Walk alone
7 Kaiser et al. [12]	M	Vesical paralysis, paresthesia, quadriparesis, bilateral facial palsy	9	Acute posterior MI	Prednisolone	Streptokinase	NO	Good return of movement and paresthesia
8 Ancillo et al. [13]	M	Paresthesia, areflexia, and quadriparesis	15	Acute inferior MI	Methylprednisolone	Anistreplase	NO	Died for ventricular arrhythmia
9 Sharma et al. [14]	M	Paresthesia, quadriparesis, dysarthria, right facial paralysis	6	ST-segment elevation in all precordial leads (V1 -V6)	Plasmapheresis	Diuretic, ACE inhibitor	NO	Walk alone
10 Ng and Stafford [15]	M	Paresthesia, quadriparesis, areflexia, dyspnoea	10	Acute ST-segment elevation anterior MI	IVIg	N/A	YES	Gradual recovery
11 Eshraghian et al. [16]	M	Paresthesia, limb weakness, dyspnoea	7	ST-elevation in V2-V4 precordial leads	IVIg	Streptokinase	YES	Four limb muscle power returning to 3 of 5
12 Kumar et al. [17]	M	Paresthesia, limb weakness, bilateral facial paralysis, dysphagia, dyspnoea	17	Acute ST-segment elevation anterior MI, the left anterior descending artery proximal 90% stenosis	IVIg	Streptokinase, angioplasty, stent implantation	YES	Good return of motor function
13 Gajjar et al. [18]	M	Autonomic disfunction, paresthesia, quadriparesis, respiratory failure	6	Acute inferior MI	Plasmapheresis	Operated for ventricular septal defect	YES	Good return of movement and respiratory
14 Aldag et al. [20]	M	Paresthesia, limb weakness, ataxia, left-sided ptosis, weakness and dysphagia, dyspnoea	5	Anterior ST-segment elevation MI, Triple-vessel coronary artery disease	Plasmapheresis, IVIg	Coronary artery bypass under cardiopulmonary bypass (CPB) with 30 hypothermia	YES	Died for cardiopulmonary arrest
15 Kumar et al. [19]	M	Respiratory failure, areflexic, quadriparesis	1	Anterior wall non-ST elevation MI, left anterior descending artery and midright coronary artery stenosis (respectively 90% and 80%)	IVIg	Off-pump coronary artery bypass graft	YES	Complete recovery
16 Our case	F	Paresthesia, quadriparesis, respiratory failure	4	ST-segment elevation in leads II、III、aVF、V3-V5, V7-V9	IVIg	PCI	YES	Died for cardiopulmonary arrest

*M* Male, *F* Female, *IVIg* Intravenous immunoglobulin, *GBS* Guillain–Barre syndrome, *MI* Myocardia infarction, *PCI* Percutaneous coronary intervention, *MV* Mechanical ventilation

**Table 2:** General characteristics and clinical outcomes of the 16 patients with GBS after MI

Varible	Total (*n* = 16)	Prognosis	
		Good (*n* = 13)	Died (*n* = 3)
Male	14(87.5)	12(92.3)	2(66.7)
female	2(12.5)	1(7.3)	1(33.3)
Clinical manifestations			
Limb weakness	15(93.7)	12(92.3)	3(100)
Quadriparesis	11(68.8)	9(69.2)	2(66.7)
Paresthesia	13(81.2)	10(76.9)	3(100)
Papilloedema	1(6.25)	1(7.7)	0
Unilateral facial paralysis	2(12.5)	2(15.4)	0
Bilateral facial paralysis	3(18.8)	3(23.1)	0
Autonomic disfunction	3(18.8)	3(23.1)	0
Dysphagia	4(25)	2(15.4)	2(66.7)
Dyspnoea	9(56.3)	7(53.8)	2(66.7)
Treatment for GBS			
Immunoglobulin	5(31.3)	3(23.1)	2(66.7)
Plasmapheresis	3(18.8)	2(15.4)	1(33.3)
Plasmapheresis + immunoglobulin	1(6.25)	0	1(33.3)
Corticosteroids	3(18.8)	2(15.4)	1(33.3)
Other	4(25)	4(30.8)	0
Treatment for MI			
Streptokinase	5(31.3)	4(30.8)	1(33.3)
Surgery	4(25)	2(15.4)	2(66.7)
Surgery + Streptokinase	2(12.5)	2(15.4)	0
Other	2(12.5)	2(15.4)	0
Nil	3(18.8)	3(23.1)	0
Type of MI			
Anterior	6(37.5)	5(38.5)	1(33.3)
Inferior	7(43.8)	6(46.2)	1(33.3)
Posterior	1(6.25)	1(7.7)	0
Anterior + inferior + posterior	1(6.25)	0	1(33.3)
Nil	1(6.25)	1(7.7)	0
ST-segment elevation MI	8(50)	7(53.8)	1(33.3)
MV	9(56.3)	7(53.8)	2(66.7)

*MI* Myocardia infarction, *GBS* Guillain–Barre syndrome, *MV* Mechanical ventilation

When patients with MI present with limb and/or respiratory muscle weakness for the first time, they should be transferred to the intensive care unit (ICU). The cause of muscle weakness needs to be identified early to select appropriate treatment. The main differential diagnoses include critical illness polyneuropathy, critical illness myopathy, critical illness neuromyopathy, and acute quadriplegic myopathy. They often occur in clinical conditions with multi-organ failure and sepsis, or after the long-term use of large doses of neuromuscular blockers and steroids, and often manifest as limb weakness, palsy, and respiratory muscle weakness. However, it has been shown that GBS can be distinguished from these disorders base on precipitating events, CSF examinations, microbiology, immunology, electrophysiological characteristics, and peripheral nerve morphology. GBS usually occurs due to infection, and in a small number of cases, trauma prior to admission to the ICU can be a precipitating event. Electrophysiological studies of patients with GBS show demyelinating polyneuropathy. Morphological studies of the nerve may suggest inflammation. Microbiology and immunology analyses show anti-GM1, anti-CD1B, IgM to C. jejuni, serology IgA, and stool culture. GBS shows a typical albumin-cytologic dissociation in CSF examinations, which can contribute to the diagnosis of GBS. In our case study, the clinical diagnosis of GBS was based on clinical symptoms, including acute tetraplegia, bulbar weakness, and respiratory failure with areflexia. Additionally, antiganglioside antibodies have been used as diagnostic serum biomarkers in clinical practice for a long time, anti-GT1a IgG antibodies and anti-GM1 IgG antibodies contribute to the diagnosis of acute motor axonal neuropathy (AMAN), and anti-GM1 IgG antibodies also can be detected in acute motor-sensory axonal neuropathy (AMSAN) [21]. Additionally, presence of serum anti-GT1a IgG antibodies, anti-GM1 IgG antibodies, and anti-GM2 IgG antibodies favoured the diagnosis of GBS. Positive ganglioside antibodies are commonly seen in GBS with prodromal infection, but whether the presence of ganglioside antibodies in GBS induced by myocardial infarction/surgery needs to be further studied. At later stages, our patient presented with complications of severe infection, rhabdomyolysis, and circulatory collapse, which predicted a poor prognosis.

**Fig. 2:**
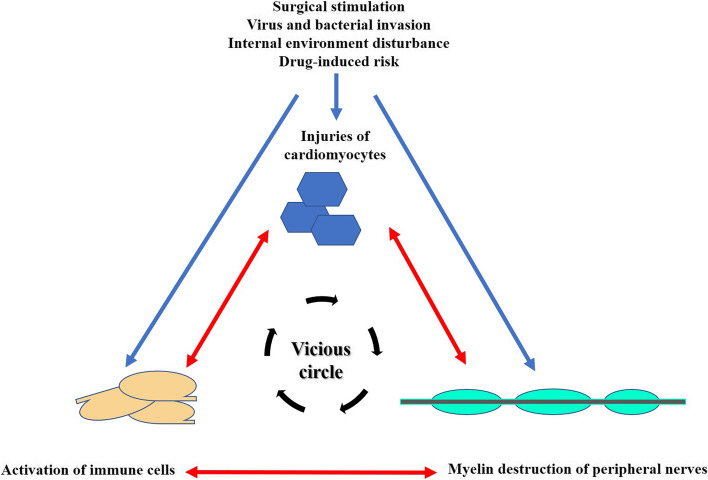
The pattern diagram of the vicious circle about immune mediated demyelination of peripheral nerves triggered by cardiac events

The causal mechanism of GBS is immune-mediated. However, the causal mechanism of GBS after MI is currently unknown. The possible trigger of GBS after MI can be explained by several hypotheses (Fig. 2). Firstly, apoptotic cardiomyocytes and stroma fragments in infarction area release damage-related mode molecules to activate the innate immune system, which then activates inflammatory signalling pathways and generates a strong inflammatory response [22]. Additionally, inflammatory immune responses in the myocardium are activated during cardiac remodelling after MI. Therefore, intracellular molecules, fragments of the extracellular matrix, and cardiac remodelling that activate the inflammatory immune response after MI may induce GBS. Secondly, thrombolytic agents are known initiators of GBS. Kumar et al. [17] reported that GBS is a potentially life-threatening rare complication of thrombolysis. In the 16 cases of GBS after MI, 7 patients were treated with streptokinase, which is a single chain polypeptide extracted from beta-haemolytic streptococci. Its protein nature makes itself antigenic in vivo and, hence, activates immunologic responses. This may be the pathophysiologic mechanism underlying the development of GBS after streptokinase treatment for MI. However, others have argued against an association between GBS and streptokinase treatment sighting the low incidence of this complication [23]. Whether streptokinase is an inducer of GBS, remains to be explored. Thirdly, surgery can imbalance the immune system and/or amplify the immune response triggered by viral infection. Given the heterogeneity of GBS triggered by surgery, it is tempting to assume that the potential mechanisms include the effects of a surgery on the cellular and humoral immune systems. Studies have shown that surgeries suppress the immune system by activating the neuroendocrine stress axis [24, 25], which prompts auto-antibodies to attack the surrounding nerves and may also amplify the immune response triggered by previous viral infections. Additionally, the release of intraoperative antigens and antibodies may induce an autoimmune response [24]. Inflammatory responses can be triggered by many factors, including cardiopulmonary bypass, ischaemia, and reperfusion injury, which are caused by heart surgery. Cardiac events may trigger a vicious cycle of immune-mediated peripheral nerve demyelination, which can be represented by a pattern diagram shown in Fig. 1. Of all the 16 cases, 6 patients underwent surgeries, including PCI in our case, which has never been reported before. We made a bold guess that MI and/or PCI may activate the immune response to induce GBS.

Plasmapheresis is designated as a Category I therapy for treating GBS by The American Society for Apheresis and is considered the most efficacious treatment. However, because of its invasive features and high cost, its usage in clinical practice is restricted. Early treatment with IVIg, which is the most practical treatment in clinical settings, is considered to be a replacement for plasmapheresis, and it reduces the duration and severity of paresis. The use of corticosteroids for GBS is controversial. Due to the small sample size, our paper can’t determine which treatment is more effective, it remains to be further explored.

There are some limitations of our case. Firstly, since the patient required continuous ventilator-assisted ventilation, brain MRI and cervical spine MRI could not be completed to more directly exclude cerebral infarction. However, the repeated brain CT showed no abnormality, and the pathological signs remained negative and the reflex decreased, all of which were more supported the diagnosis of GBS. Secondly, due to the critical condition of the patient and the refusal of the patient's family, lumber puncture and NCS could not be completed, we detected the serum anti-ganglioside antibodies to support the diagnosis.

In conclusion, MI and/or PCI may be a trigger of immune-mediated response and may cause severe complications. The clinical manifestations of GBS are diverse. Clinician should be alert to GBS after MI and/or PCI.

## Supplementary Information

**Additional file 1: Supplementary Figure S1.** Angiograms of the right coronary artery. Initial angiogram showed multiple branches and diffuse lesions in the middle segment of the right crown, with the most severe stenosis reaching 85%, while those taken after PCI show restoration of flow.

## Data Availability

All data generated or analysed during this study are included in this published article.

## Abbreviations

M: Male
F: Female
IVIg: Intravenous immunoglobulin
GBS: Guillain–Barre syndrome
MI: Myocardia infarction
CFS: Cerebrospinal fluid
NCS: Nerve conduction studies
PCI: Percutaneous coronary intervention
MV: Mechanical ventilation
CMV: Cytomegalovirus
ICU: Intensive care unit
