# Correction to: Guillain–Barre syndrome after myocardial infarction: a case report and literature review

**DOI:** 10.1186/s12872-023-03299-4

**Published:** 2023-05-17

**Authors:** Pu-yuan Wen, Xian-wen Chen, Min Zhang, Wen-zheng Chu, Hong-liang Wu, Chao Ren

**Affiliations:** 1 Department of Neurology, Affiliated Yantai Yuhuangding Hospital of Qingdao University, Yantai, 264000 Shangdong China; 2 Department of Neurology, The First Affiliated Hospital of Anhui Medical University, Hefei, 230000 Anhui China

Following publication of the original article [1], the affiliation of the authors has been updated and it will read as follows:

Pu-yuan Wen^1,2†^, Xian-wen Chen ^2†^, Min Zhang^1^, Wen-zheng Chu^1^, Hong-liang Wu^1^, Chao Ren^1*^

The original article has been corrected.

