# Supplementary material for: Guillain–Barre syndrome after myocardial infarction: a case report and literature review
Source: BMC Cardiovasc Disord. 2023 May 1;23:226. doi: 10.1186/s12872-023-03261-4 (PMC10150548; doi:10.1186/s12872-023-03261-4)
Supplement: Supplementary file 1 — Additional file 1: Supplementary Figure S1. Angiograms of the right coronary artery. Initial angiogram showed multiple branches and diffuse lesions in the middle segment of the right crown, with the most severe stenosis reaching 85%, while those taken after PCI show restoration of flow. [file 12872_2023_3261_MOESM1_ESM.pdf]

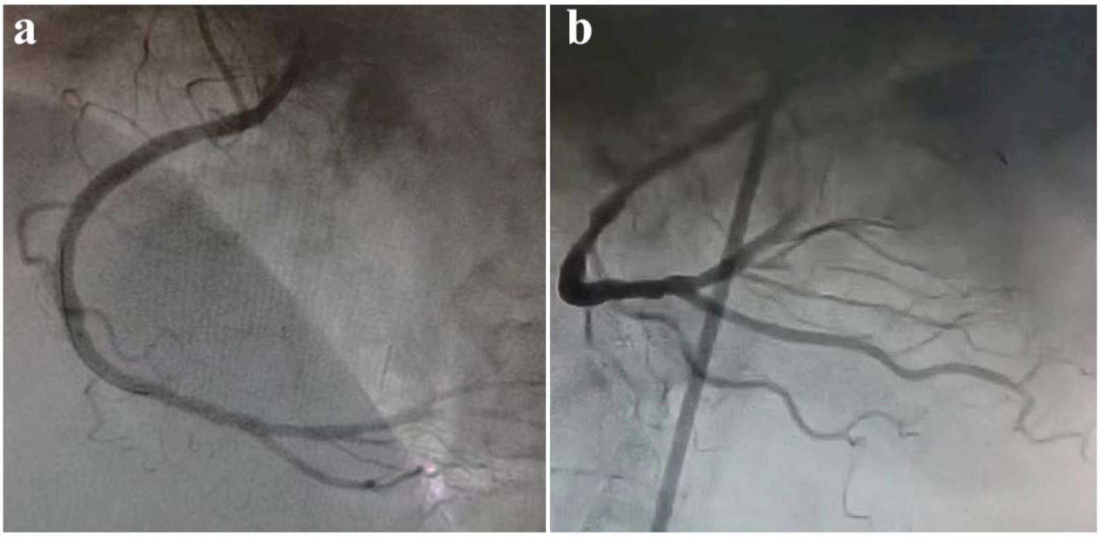

Supplementary Figure S1. Angiograms of the right coronary artery. Initial angiogram showed multiple branches and diffuse lesions in the middle segment of the right crown, with the most severe stenosis reaching 85%(a), while those taken after PCI show restoration of flow.
